# Service evaluation of faecal immunochemical testing and anaemia for risk stratification in the 2‐week‐wait pathway for colorectal cancer

**DOI:** 10.1002/bjs5.50131

**Published:** 2019-01-28

**Authors:** C. Chapman, J. Bunce, S. Oliver, O. Ng, A. Tangri, R. Rogers, R. F. Logan, D. J. Humes, A. Banerjea

**Affiliations:** ^1^ Eastern Hub, Bowel Cancer Screening Programme Nottingham University Hospitals NHS Trust Nottingham UK; ^2^ Nottingham Colorectal Service Nottingham University Hospitals NHS Trust Nottingham UK; ^3^ National Institute for Health Research Nottingham Digestive Diseases Biomedical Research Centre, Nottingham University Hospitals NHS Trust Nottingham UK; ^4^ Nottingham City Clinical Commissioning Group University of Nottingham Nottingham UK; ^5^ Division of Epidemiology and Public Health, School of Medicine, University of Nottingham Nottingham UK

## Abstract

**Background:**

New national guidance on urgent referral for investigation of colorectal cancer included faecal occult blood testing in 2015. A service evaluation of faecal immunochemical testing (FIT) and anaemia as risk stratification tools in symptomatic patients suspected of having CRC was undertaken.

**Methods:**

Postal FIT was incorporated into the colorectal cancer 2‐week wait (2WW) pathway for all patients without rectal bleeding in 2016. Patients were investigated in the 2WW pathway as normal, and outcomes of investigations were recorded prospectively. Anaemia was defined as a haemoglobin level below 120 g/l in women and 130 g/l in men.

**Results:**

FIT kits were sent to 1106 patients, with an 80·9 per cent return rate; 810 patients completed investigations and 40 colorectal cancers were diagnosed (4·9 per cent). FIT results were significantly higher in patients with anaemia (median (i.q.r.) 4·8 (0·8–34·1) *versus* 1·2 (0–6·4) μg Hb/g faeces in those without anaemia; *P* < 0·001). Some 60·4 per cent of patients (538 of 891) had a result lower than 4 μg haemoglobin (Hb) per g faeces (limit of detectability), and 69·7 per cent (621 of 891) had less than 10 μg Hb/g faeces. Some 60 per cent of patients with colorectal cancer had a FIT reading of 150 μg Hb/g faeces or more. For five colorectal cancers diagnosed in patients with a FIT value below 10 μg Hb/g faeces, there was either a palpable rectal mass or the patient was anaemic. A FIT result of more than 4 μg Hb/g faeces had 97·5 per cent sensitivity and 64·5 per cent specificity for a diagnosis of colorectal cancer. A FIT result above 4 μg Hb/g faeces and/or anaemia had a 100 per cent sensitivity and 45·3 per cent specificity for colorectal cancer diagnosis.

**Conclusion:**

FIT is most useful at the extremes of detectability; strongly positive readings predict high rates of colorectal cancer and other significant pathology, whereas very low readings in the absence of anaemia or a palpable rectal mass identify a group with very low risk. High return rates for FIT within this 2WW pathway indicate its acceptability.

## Introduction

Colorectal cancer is common, with over 41 000 new diagnoses made annually in the UK^1^. In 2000, guidelines for urgent referral on a 2‐week‐wait (2WW) pathway were introduced, with the aim of early diagnosis by promoting rapid assessment and investigation^2^. However, the cancer detection rate within this pathway remains below 10 per cent and more cancers are diagnosed in patients referred via routine pathways^3^, ^4^, ^5^, ^6^. One‐third of patients referred on a 2WW pathway have no detectable pathology, and a further third have no pathology that mandates intervention or treatment. More recently, National Institute for Health and Care Excellence (NICE) guidance (NG12) updated and broadened the referral criteria, with a benchmark for investigation of any symptom with a positive predictive value for cancer greater than 3 per cent^7^. Colonoscopy remains the standard investigation of the whole colon, and is used to investigate 85–90 per cent of patients referred to the Nottingham 2WW pathway^8^. However, it is expensive and invasive, with recognized complications, and nationally the pressure on endoscopy services is growing to potentially unsustainable levels. Flexible sigmoidoscopy^9^ and CT colonography^10^ present alternatives for investigation of low‐risk patients, but ideally in the context of safe and reliable risk stratification within the 2WW pathway^11^.

The Bowel Cancer Screening Programme has achieved significant positive stage migration towards early cancer at diagnosis, and the conversion rate from positive occult blood testing has been shown generally to exceed the detection rate seen in 2WW pathways^8^. In this context, new NICE guidance (NG12) that broadened referral criteria and aimed to ‘rule in’ more low‐risk patients by utilizing occult blood testing was seen as a double‐edged sword in many quarters. NG12 did not specify the type of occult blood testing that should be used; however, studies have suggested that faecal immunochemical testing (FIT) might be useful in symptomatic pathways^12^, ^13^, ^14^ and potentially more accurate than symptoms alone in predicting which patients need investigation for cancer^15^, ^16^, ^17^.

In Nottingham, 2WW referrals are not rejected as only the referring general practitioner (GP) may retract or downgrade a referral, in keeping with guidance on cancer waiting times^18^. Therefore, simple risk stratification to improve clinical effectiveness within the 2WW pathway has been a long‐term aim. Data monitoring of the straight‐to‐test (STT) colonoscopy pathway was established in August 2014 to ensure clinical governance and clinical effectiveness^8^. At that time, haemoglobin (Hb) concentration was introduced as a mandatory field for all new 2WW referrals, but compliance was poor. Anaemia was found to identify higher‐risk patients in all patient groups, and change in bowel habit without anaemia has a predictive value for colorectal cancer of less than 3 per cent in the Nottingham 2WW pathway^19^.

In December 2015, as a result of NG12, a collaborative ‘Getting FIT’ working group was established with local GPs and commissioners, the Bowel Cancer Screening Hub and Nottingham Colorectal Service. In September 2016, Nottingham City, Nottingham West, Nottingham North and East, and Rushcliffe Commissioning Groups commissioned Getting FIT, thereby incorporating FIT as a triage tool in the 2WW pathway. Approximately 1000 tests were commissioned to assess the feasibility of incorporating FIT into local symptomatic pathways for colorectal cancer.

The aim of the present study was to evaluate anaemia and faecal haemoglobin (fHb) levels as risk stratification tools in a 2WW pathway, and to assess FIT within an operational urgent colorectal cancer pathway in England.

## Methods

### Referral pathway service development

The STT pathway has been described in detail previously^8^. In brief, every 2WW referral received is vetted jointly by a colorectal nurse practitioner (CNP) and a consultant colorectal surgeon daily. Patients considered suitable for STT are then contacted by telephone and assessed for suitability to ensure patient safety and appropriateness. An alternative outcome from the vetting process is review in clinic by either a CNP or a consultant. Patients failing the telephone vetting process (for example not contactable, unable to answer questions or fitness difficult to determine remotely) are diverted to a CNP‐led clinic. Patients deemed frail, elderly or co‐morbid during vetting are directed to a consultant clinic, but may undergo CT before appointment. Referrals are logged prospectively in a database as part of the clinical governance arrangements established to evaluate STT and consistent with Cancer Outcomes and Services Dataset (COSD) requirements and best practice guidance for STT pathways.

### Service modification

The STT vetting process was modified to highlight patients referred on the 2WW pathway who should be sent a FIT kit. All patients who were not referred with rectal bleeding were considered for FIT assessment. The standard 2WW/STT process was not interrupted, and FIT test results were not used to determine the vetting outcome. Markedly raised FIT results in returned kits were, however, used to prioritize patients for expeditious investigation.

### Data sources and cohorts

All patients referred under the 2WW pathway from primary care for suspected colorectal cancer between 6 September 2016 and 31 August 2017 were included, and all outcomes were censored on 22 September 2017. Patient demographics, referral data, vetting outcomes, FIT results and clinical outcomes for all 2WW referrals were recorded on a NUhCLEUS software system (developed locally by Nottingham Colorectal Service). The data set is maintained by specialist nurses and a dedicated audit clerk at the Colorectal Service at Nottingham University Hospitals NHS Trust. The results were also monitored continuously for safety by project leads. Patients referred with rectal bleeding were excluded from FIT stratification.

### Faecal immunochemical testing process

All patients referred without rectal bleeding were posted a faecal sample collection device (OC‐Sensor™; Eiken Chemical Company, Tokyo, Japan) within 2 days of the 2WW referral being received. The device was prelabelled with the patient's name, National Health Service (NHS) number, a unique laboratory ID number and a space to add the sample date. An instruction leaflet for using the sampling device, a letter outlining the purpose of the test and clarifying that the results would not be used for diagnostic purposes in isolation, and a prepaid first‐class return envelope were also included. Participants were asked to sample their faeces according to instructions, date the sampling device, and return it to the laboratory within 14 days of receipt of the letter.

All returned samples were logged at the receiving laboratory and analysed for fHb using the automated OC‐Sensor™ Diana (Eiken Chemical Company) according to manufacturer's protocols, alongside fHb controls. The analyser was calibrated once a month, and two levels of control were validated at the beginning and end of each run. Returned samples were stored in a refrigerator at 4 °C upon arrival, until analysis. All samples were analysed within 1 week of receipt.

Analyses were carried out in laboratories located at the Eastern Bowel Cancer Screening Hub, Nottingham, UK. These laboratories are accredited by the UK Accreditation Service (ISO 15189) and also take part in UK National External Quality Assessment Service external quality‐assessment schemes.

### Result notifications

The results of analyses of all returned kits were forwarded to the STT team on a weekly basis. 2WW FIT results were reviewed continuously by the STT team. Patients with readings of 150 μg Hb per g faeces or above were prioritized for additional contact, early appointments or expedited investigation. This cut‐off was preselected to be equivalent to a positive guaiac faecal occult blood test, the only alternative in clinical practice at the time. Clinicians could access the FIT results if they chose to, and also receive notification when the initial clinical decision was not to undertake whole‐colon investigation although the FIT result was 150 μg Hb/g faeces or more. Further investigation was then at the discretion of the clinician and their patient.

### Data analysis

#### *Exposure and co‐variables*

Patients were considered to be anaemic, as defined by the WHO^20^, when their Hb level was less than 120 g/l for women or 130 g/l for in men, based on the most recent Hb estimation within 3 months before referral. Patients without a recent Hb assessment before referral were unclassified for anaemia. Age was categorized as: 18–49, 50–59, 60–69, 70–79 and 80 years or above. fHb levels were determined and categorized as: above 150, 10–149·9, 4–9·9 and less than 4 μg Hb/g faeces. The lower cut‐offs were chosen as 10 μg Hb/g faeces was recommended by NICE guidance on quantitative FIT (diagnostic guidance DG30)^16^, and 4 μg Hb/g faeces was the limit of reliable detectability on the analyser platform.

#### *Outcome definition*

Colorectal cancer and other diagnoses were determined from investigation outcomes by reviewing endoscopy, radiology and histology reports, and clinic letters.

Other significant bowel pathology was defined as inflammatory bowel disease, complicated diverticular disease, adenomas requiring endoscopic follow‐up (1 polyp greater than 1 cm in size or more than 3 confirmed adenomas), and suspicious polyps and early colorectal cancer (SPECC) lesions requiring multidisciplinary team assessment and urgent removal (if proven to be malignant they were included in the colorectal cancer group). These conditions were chosen as diagnoses encountered in the 2WW pathway that usually require further intervention in secondary care.

### Statistical analysis

Data were assessed for normality using histograms and the Shapiro–Wilks test. Comparisons were made between continuous variables using Student's *t* test when normally distributed and the Mann–Whitney *U* test when not normally distributed, or with ANOVA to compare across multiple groups. Categorical data were summarized using frequencies and percentages. Comparisons were made between categorical data with χ^2^ tests. Sensitivity, specificity, positive predictive (PPV) and negative predictive (NPV) values were calculated along with 95 per cent c.i. for each cut‐off of FIT. A further analysis calculated these for the appropriate FIT cut‐off and/or a diagnosis of anaemia, when known. Statistical analysis was performed using Stata® version 15 (StataCorp, College Station, Texas, USA). *P* < 0·050 was considered significant.

### Funding

The project was commissioned locally to enable and evaluate access to FIT for local GPs. The cost of 1000 FIT tests was accounted for in the initial business case. All four local clinical commissioning groups (CCGs: Nottingham City, Nottingham North and East, Nottingham West and Rushcliffe) approved and jointly funded this service evaluation project. The cost of each FIT test was agreed at £15·00 (approximately €17, exchange rate 30 November 2018) per sample to CCGs, including postage, analysis and administration costs.

## Results

**Figure 1:**
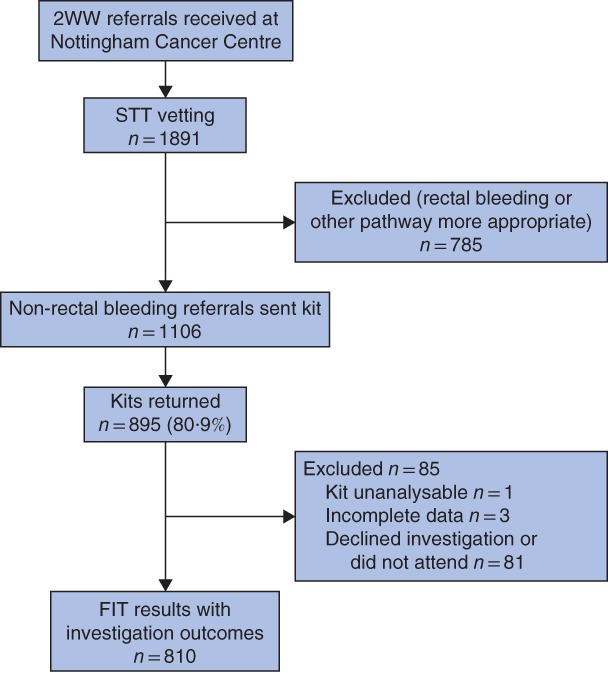
Flow diagram of patients in the ‘Getting FIT’ pathway, from referral to analysis of results 2WW, 2‐week‐wait; STT, straight‐to‐test; DNA, did not attend; FIT, faecal immunochemical test.

During the study period, 1891 2WW referrals were vetted by the STT team (*Fig*. 1). Some 1106 referrals were deemed suitable for FIT and were sent kits, 895 OC‐Sensor™ kits were returned (80·9 per cent), three patients had incomplete data and one kit was unanalysable. The median kit return time was 7 (range 2–79) days, and 93·9 per cent of kits (840 of 895) were returned within 14 days. The median age of those referred was 71·7 (i.q.r. 62·6–79·3) years,

### Faecal immunochemical test readings

Of the 895 returned kits, 891 yielded analysable OC‐Sensor™ results. Median levels of fHb were higher in men than in women (2·4 (i.q.r. 0–23·2) *versus* 1·8 (0–14·8) μg Hb/g faeces respectively; *P* = 0·059), but the difference was not statistically significant despite a significantly higher rate of colorectal cancer in men than women across the cohort (6·8 *versus* 2·6 per cent; *P* < 0·003). Median levels of fHb increased with age, but not significantly so (*P* = 0·931) (*Table* 1). Colorectal cancer was significantly associated with age (*P* = 0·003).

**Table 1:** Demographics and faecal immunochemical test levels

	2WW referrals (*n* = 891)	FIT result (μg Hb/g faeces)†
**Sex**		
M	395 (44·3)	2·4 (0–23·2)
F	496 (55·7)	1·8 (0–14·8)
**Age (years)**		
0–59	173 (19·4)	0·4 (0–4·2)
60–79	512 (57·5)	2·2 (0–15·3)
≥ 80	206 (23·1)	4·8 (1·2–35·0)

* Values in parentheses are percentages;

† values are median (i.q.r.). 2WW, 2‐week‐wait; FIT, faecal immunochemical test; Hb, haemoglobin.

In total, 538 (60·4 per cent) returns yielded a level lower than 4 μg Hb/g faeces (limit of detectability in the laboratory) and 621 (69·7 per cent) were below 10 μg Hb/g faeces. Seventy‐two patients (8·1 per cent) had a level of 150 μg Hb/g faeces or above.

### Faecal immunochemical testing and diagnosis

During the study, 810 patients returned an analysable FIT kit and also underwent investigation on the 2WW pathway; 9·5 per cent (85 of 895) declined investigation, did not attend or had missing data.

Clinical outcomes of patients referred on the 2WW pathway are summarized in *Table* 2. Colorectal cancers demonstrated the highest median FIT readings, yielding significantly higher FIT results than some other diagnosis groups (normal, diverticular disease, piles and microscopic colitis; *P* < 0·001), but not compared with results for other significant bowel pathology.

**Table 2:** Final diagnosis after investigation and faecal immunochemical test results

Diagnosis	2WW referrals (*n* = 810)*	FIT result(μg Hb/g faeces)†
Normal	476 (58·8)	1·2 (0–5·2)
Cancer	40 (4·9)	223 (34·8–1334·7)
Other cancer	18 (2·2)	15·4 (2·0–170·2)
High‐risk adenoma	45 (5·6)	20·6 (2·8–83·2)
Low‐risk adenoma‡/benign UGI lesion	168 (20·7)	2 (0–11·9)
SPECC	10 (1·2)	127 (17·4–898·6)
Colitis	22 (2·7)	42·2 (1·6–314·8)
Microscopic colitis	29 (3·6)	2 (0–6·2)
Complicated diverticular disease	2 (0·2)	17·7 (14–21·4)

* Values in parentheses are percentages;

† values are median (i.q.r.).

‡ Deemed not to require further endoscopic follow‐up.

§ Significant polyps or early colorectal cancer (SPECC) include lesions considered suspicious for cancer, discussed at a specific multidisciplinary team meeting with a view to consideration of urgent removal. 2WW, 2‐week‐wait; FIT, faecal immunochemical test; Hb, haemoglobin; UGI, upper gastrointestinal tract.

### Faecal immunochemical testing and colorectal cancer

During the study, 40 colorectal cancers (23 left‐sided, 17 right‐sided) were diagnosed in the population that returned a FIT kit and were investigated. The majority (60 per cent) had a FIT reading of 150 μg Hb/g faeces or more. Two patients had originally declined investigation but reconsidered when counselled with their FIT results. A further two patients initially underwent CT with intravenous contrast as per local protocol for older frailer patients^8^; the scans were reported as normal. FIT results of 150 μg Hb/g faeces or above prompted further investigation and demonstrated colorectal cancer in both patients. Eleven patients with colorectal cancer had FIT readings between 10·0 and 149·9 μg Hb/g faeces. Five colorectal cancers were diagnosed in patients with a FIT result below

10 μg Hb/g faeces, but all had either a palpable rectal mass (not mentioned in the referral) or were anaemic. No colorectal cancers were detected with a FIT reading below 2 μg Hb/g faeces, although the reliability of the platform at this level of fHb could not be validated in the laboratory.

Right‐sided cancers were associated with a significantly lower FIT result than left‐sided lesions (median 41·6 (i.q.r. 11·2–406·8) *versus* 286·8 (142–5076·8) μg Hb/g faeces respectively; *P* = 0·030). Inclusion of SPECC lesions did not alter the significance of this comparison of laterality, with right colorectal cancers and SPECC demonstrating lower median fHb levels (61·2 (i.q.r. 15·8–403·4) *versus* 329·7 (71·8–1705·0) μg Hb/g faeces; *P* = 0·027).

FIT results of 4 μg Hb/g faeces or above demonstrated 97·5 per cent sensitivity and 64·5 per cent specificity for the diagnosis of colorectal cancer (*Table* 3). FIT had a NPV of 97·8 per cent and PPV of 35·8 per cent for colorectal cancer using a cut‐off of 150 μg Hb/g faeces (*Table* 3). The NPV and sensitivity rose to over 99 per cent with lower cut‐off values.

**Table 3:** Sensitivity, specificity, positive predictive and negative predictive values for colorectal cancer with faecal immunochemical test cut‐off values alone or with anaemia

	Sensitivity (%)	Specificity (%)	PPV (%)	NPV (%)
**FIT cut‐off (μg Hb/g faeces)**				
≥ 4·0	97·5 (86·8, 99·9)	64·5 (61·1, 67·9)	12·5 (9·0, 16·7)	99·8 (98·9, 100)
≥ 10·0	87·5 (73·2, 95·8)	73·5 (70·2, 76·6)	14·6 (10·4, 19·8)	99·1 (98·0, 99·7)
≥ 150·0	60 .0 (43·3, 75·1)	94·4 (92·6, 95·9)	35·8 (24·5, 48·5)	97·8 (96·5, 98·8)
**FIT cut‐off (μg Hb/g faeces) and/or anaemia**				
≥ 4·0	100 (90·5, 100)	45·3 (41·6, 49·0)	8·6 (6·1, 11·6)	100 (98·9, 100)
≥ 10·0	97·3 (85·8, 99·9)	51·7 (47·9, 55·4)	9·3 (6·6, 12·7)	99·7 (98·5, 100)
≥ 150·0	86·5 (71·2, 95·5)	61·3 (57·7, 64·9)	10·3 (7·12, 14·2)	98·9 (97·4, 99·6)

Values in parentheses are 95 per cent confidence intervals. PPV, positive predictive value; NPV, negative predictive value; FIT, faecal immunochemical test; Hb, haemoglobin.

### Faecal immunochemical testing and anaemia

**Figure 2:**
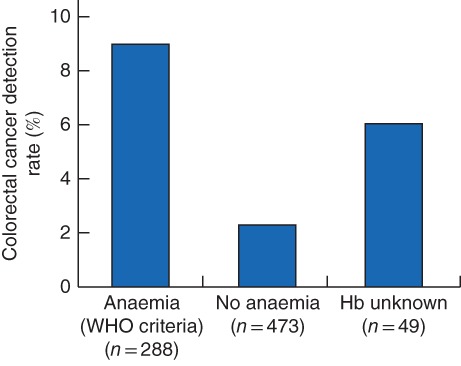
Colorectal cancer detection rate by presence of anaemia as defined by WHO criteria at referral Hb, haemoglobin.

In total, 761 patients had a valid Hb assessment for classification of anaemia, with 288 (37·8 per cent) presenting with anaemia. FIT results were significantly higher in patients with anaemia (median (i.q.r.) 4·8 (0·8–34·1) *versus* 1·2 (0–6·4) μg Hb/g faeces in those without anaemia; *P* < 0·001. The colorectal cancer detection rate was higher in anaemic (26 of 288, 9·0 per cent) than in non‐anaemic patients (11 of 473, 2·3 per cent) (*P* = 0·001) (*Fig*. 2). Three of the 49 patients (6 per cent) without a recent Hb test result available were diagnosed with colorectal cancer. The sensitivity and specificity of each FIT cut‐off value and/or anaemia are shown in *Table* 3.

### Faecal immunochemical testing and referral symptoms

The overall colorectal cancer detection rate in patients referred with ‘change in bowel habit’ alone was 3·0 per cent. FIT results discriminated low‐risk groups with a 0 per cent colorectal cancer detection rate (fHb below 4·0 μg Hb/g faeces) and 2·1 per cent detection rate (fHb between 4·0 and 9·9 μg Hb/g faeces), from high‐risk patients with a colorectal cancer detection rate of 34·6 per cent (fHb 150 μg Hb/g faeces or more) (*Table* 4). FIT vales were similar in most symptom groups other than iron deficiency anaemia, where the colorectal cancer detection rate was 1·2 per cent in patients with less than 4·0 μg Hb/g faeces but 15·0 per cent in those with results between 4·0 and 9·9 μg Hb/g faeces.

**Table 4:** Colorectal cancer detection rates in different symptom groups stratified by faecal immunochemical test results

			Colorectal cancer detection rate stratified by FIT cut‐off (μg Hb/g faeces) (%)			
Referral symptom	% of referrals*	Overall risk of colorectal cancer (%)	< 4·0	4·0–9·9	10·0–149·9	≥ 150·0
Combined symptoms†	12·2	6·1	0	0	5·2	41·7
IDA	22·1	8·4	1·2	15·0	7·2	30·4
CIBH alone	58·2	3·0	0	2·1	4·5	34·6
Abdominal mass	3·5	10·7	0	0	14·3	66·7
Rectal mass	4·0	6·3	0	0	50·0	33·3

* Total of 795 referrals with 15 excluded as referral criteria were unclear.

† Any combination with more than one symptom. FIT, faecal immunochemical test; Hb, haemoglobin, IDA, iron deficiency anaemia; CIBH, change in bowel habit.

### Faecal immunochemical testing and significant bowel pathology

Significant bowel pathology was diagnosed in 108 patients (13·3 per cent) (*Table* 2). The performance characteristics of FIT alone, and FIT result and/or anaemia, for significant bowel pathology are shown for different cut‐off values (*Table* 5). FIT alone had a PPV of 53·7 per cent at levels of 150 μg Hb/g faeces or above.

**Table 5:** Sensitivity, specificity, positive predictive and negative predictive values for significant bowel pathology with faecal immunochemical test cut‐off values alone or with anaemia

	Sensitivity (%)	Specificity (%)	PPV (%)	NPV (%)
**FIT cut‐off (μg Hb/g faeces)**				
≥ 4·0	81·4 (72·3, 88·6)	67·3 (63·7, 70·8)	25·3 (20·6, 30·5)	96·4 (94·3, 97·8)
≥ 10·0	75·3 (65·5, 83·5)	76·7 (73·4, 79·8)	30·5 (24·8, 36·8)	95·8 (93·8, 97·3)
≥ 150·0	37·1 (27·5, 47·5)	95·7 (93·9, 97·0)	53·7 (41·1, 66·0)	91·8 (89·6, 93·7)
**FIT cut‐off (μg Hb/g faeces) and/or anaemia**				
≥ 4·0	87·6 (79·0, 93·7)	47·2 (43·3, 51·0)	18 (14·5, 22·0)	96·6 (94·1, 98·3)
≥ 10·0	86·5 (77·6, 92·8)	54 (50·2, 57·8)	19·9 (16·1, 24·3)	96·8 (94·5, 98·3)
≥ 150·0	68·5 (57·8, 78)	62·6 (58·9, 66·3)	19·6 (15·3, 24·4)	93·8 (91·1, 95·8)

Values in parentheses are 95 per cent confidence intervals. PPV, positive predictive value; NPV, negative predictive value; FIT, faecal immunochemical test; Hb, haemoglobin.

## Discussion

This study demonstrates that it is possible to stratify risk of a diagnosis of colorectal cancer within the 2WW pathway by incorporating simple objective measures such as anaemia and fHb levels, alongside symptoms and demographics. FIT appears to be most useful at the extremes of the scale, and is of practical value within the 2WW pathway. High FIT levels identified a group with a greater than 30 per cent risk of colorectal cancer, with four patients called back for additional investigation in the light of this result – diagnoses that would have been missed in the standard STT pathway. Arguably, use of FIT outperformed routine clinical practice. Low or undetectable levels might help to avoid unnecessary urgent investigation in more than half of the referred population.

The combination of FIT and anaemia is more sensitive than FIT alone, and achieves a sensitivity of 100 per cent when compared with standard 2WW protocols in this cohort. Anaemia is a well recognized indicator of risk in primary care for colorectal cancer^21^, ^22^, ^23^, but this study emphasizes the value of anaemia, with or without evidence of iron deficiency, as a risk stratification tool in a local diagnostic pathway for colorectal cancer^19^. However, the increased risk demonstrated with respect to anaemia is specific to symptomatic patients and not the general population. The data also confirm that a postal system for adoption of FIT is viable, with good uptake and acceptable time frames for kit return.

NICE evaluation has suggested a cut‐off of 10 μg Hb/g faeces for low‐risk patients^16^, but symptomatic patients may have colorectal cancer despite fHb readings below this level: 12·5 per cent of all colorectal cancers detected in the present cohort. This study population includes higher‐risk patients than the low‐risk patients referred to in the guidance. However, these findings are in keeping with ongoing evaluations in Dundee^13^, where the cut‐off of 10 μg Hb/g faeces has also missed colorectal cancer in patients, but all were found to have iron deficiency anaemia.

Change in bowel habit is a subjective referral symptom, and FIT has clear value for stratifying risk in this group of patients. FIT appears less useful in iron deficiency anaemia, but could still have value in guiding the choice of first test. For example, a FIT reading of less than 4 μg Hb/g faeces, with a colorectal cancer detection rate of 1·2 per cent, might prompt CT colonography instead of colonoscopy. This study excluded use of FIT in patients with rectal bleeding, but other studies have shown that undetectable blood on FIT has a reliable NPV^13^, ^14^. However, exclusion of patient with rectal bleeding identifies a high‐risk group with a PPV of 35·8 per cent for colorectal cancer and 53·7 per cent for significant bowel pathology. This is potentially more important than the ‘rule out’ functions of FIT. FIT should not be considered a stand‐alone test, but may be combined effectively with other objective criteria that can be complementary in 2WW pathways.

The incorporation of FIT into clinical practice poses challenges in the context of 2WW pathways. FIT is ideally a triage tool for primary care, but complex algorithms are difficult to roll out and ‘safety‐netting’ for GPs is vital. Scoring systems, such as the FAST (faecal haemoglobin concentration, age and sex test) score^24^, which incorporate FIT alongside demographics, may be the solution. Anaemia provides additional value in such scoring systems; thrombocytosis may also be of value^25^. Easier access to FIT may also allow diversion of large numbers of patients currently diagnosed with colorectal cancer from routine referral pathways on to 2WW pathways. This may have a significant impact on NHS services struggling with political targets. It would also be a route to creating the endoscopy capacity that enables a screening programme using FIT at its most effective threshold. If such access yields an overall stage migration towards early cancer detection, improved clinical outcomes and survival benefits may follow.
